# An out-of-lab trial: a case example for the effect of intensive exercise on rhythms of human clock gene expression

**DOI:** 10.1186/1740-3391-11-10

**Published:** 2013-09-03

**Authors:** Akihiko Okamoto, Takuro Yamamoto, Ritsuko Matsumura, Koichi Node, Makoto Akashi

**Affiliations:** 1 The Research Institute for Time Studies, Yamaguchi University, 1677-1 Yoshida, Yamaguchi 753-8511, Japan; 2 Medical Technology Research Laboratory, Medical Business Unit, R&D Div, Sony Corporation, 1-5-45 Yushima, Bunkyo-ku, Tokyo 113-8510, Japan; 3 Department of Cardiovascular Medicine, Saga University, 5-1-1 Nabeshima, Saga 849-8501, Japan

## Abstract

**Background:**

Although out-of-lab investigation of the human circadian clock at the clock gene expression level remains difficult, a recent method using hair follicle cells might be useful. While exercise may function as an entrainment cue for circadian rhythms, it remains unclear whether exercise affects human circadian clock gene expression.

**Methods:**

Efforts to observe apparent effects of exercise on clock gene expression require that several specific conditions be met: intense exercise should be habitually performed at a relatively uncommon time of day over an extended period; and any relative phase shift thereby observed should be validated by comparison of exercise and no-exercise periods. Wake-up and meal times should be kept almost constant over the experimental period. The present study was conducted using a professional fighter who met these strict criteria as subject. Facial hair samples were collected at 4-h intervals around the clock to ascertain rhythms of clock gene expression.

**Results:**

During a period in which nighttime training (from 20:00 to 22:00) was habitually performed, circadian clock gene expression was phase-delayed by 2 to 4 h compared with that during a no-exercise period. Maximum level and circadian amplitude of clock gene expression were not affected by the nighttime training.

**Conclusion:**

Our trial observations illustrate the possibility that heavy physical exercise might strongly affect the circadian phase of clock gene expression. Exercise might be therefore effective for the clinical care of circadian disorders. The results also suggest that athletes may require careful scheduling of heavy physical exercise to maintain normal circadian phase and ensure optimal athletic performance.

## Background

The circadian clock enables approximately 24-h rhythms in gene expression, which in turn leads to circadian oscillation in diverse physiological processes and thereby allows living organisms to adapt to the earth’s rotation. Investigation of the circadian clockwork requires examination of circadian clock gene expression, since the core clock is composed of cell-autonomous transcriptional feedback loops [1,2]. The clock phase is adjusted by external cues, and then synchronized with the external light/dark cycle [3,4]. Light is well known as a strong environmental cue for circadian resetting, while exercise is also thought to play a role for phase adjustment [5]. However, the effects of exercise on the phase of human clock gene expression rhythms remain unreported.

As evidenced by the correlation between circadian dysfunction and the incidence of pathological diseases such as sleep disorders, metabolic syndromes, cardiovascular diseases, mood disorders and cancer [6-10], new strategies for monitoring clock gene expression in humans are necessary for preventing circadian rhythm-related diseases [11,12]. In addition to the medical evaluation of circadian rhythm disorders, basic research on shift work and jet lag and chronopharmacological applications also require effective strategies for studying human clock gene expression. However, the lack of an established method for evaluating circadian clock gene expression has impeded the progress of study of human circadian rhythms.

Although melatonin measurement has been traditionally adopted and serum metabolites have been recently utilized as circadian markers, these are likely affected by light- or feeding-induced masking, and accordingly require a controlled environment inside the laboratory. More fundamentally, investigation of the circadian pacemaker requires the examination of circadian clock gene expression, since the core clock is composed of cell-autonomous transcriptional feedback loops. Our recently established method using hair follicle cells might solve these issues and promote out-of-lab investigation of the human clock, albeit that it still requires technical improvement [13].

Several experimental conditions may enable observation of an apparent exercise effect on clock gene expression: first, the exercise should not be performed during daytime but rather at a relatively uncommon time of day; second, habitual and intensive exercise over a long duration may be required to detect discernible effects; third, comparison between exercise and no-exercise periods will enable confirmation of exercise-induced phase shift; and fourth, wake-up and meal times should be kept almost constant over the experimental period to reduce the possibility that the data obtained result from factors other than exercise. We found a professional fighter who met these criteria to participate in this study.

## Materials and methods

### Subject information

The volunteer was a healthy male professional fighter, who was aged 34 years and weighed 92 kg at the time of sampling. His caloric intake remained largely unchanged throughout the experimental period. During the exercise period, he underwent intense training from 20:00 to 22:00 (total 2 h exercise per day), but no intense exercise (total 0 h exercise per day) during the no-exercise period from July 16 to August 31. The self-reported lifestyle habits (sleeping and eating) of the subject remained constant during the experimental period. Although we were unable to monitor the subject’s light exposure, he was under regular dark conditions at bedtime.

### Collection of hair follicle cells

Wake-up time and meal times were set based on the lifestyle habits of the subject. The subject was asked to refrain from consuming excess alcohol, eating excessive snacks, and taking long naps during sampling days. Hair follicle cells were collected by pulling the root of facial hair, and quickly soaked in dissolution buffer (QuantiGene Veritus). The volunteer collected facial hair follicles by himself outside the laboratory (home, office or training center) every four hours around the clock (at 12:00, 16:00, 20:00, 24:00, 4:00, 8:00, 12:00). Sampling during the night was performed under desktop light and finished within five minutes in order to minimize light-induced effects. The cells were stored at −20°C until measurement. At each sampling point, about five facial hair roots were required. While there may be individual and racial differences in yield, hairs from the tip of the chin tend to yield a greater amount of RNA. The present study was approved by the Ethical Review Board of Saga University, and informed consent was obtained from the subject.

### mRNA determination

RNA determination was performed using branched DNA probes (QuantiGene Veritus), as reported previously [13]. The probes were designed by the company, and their sequences are not revealed. With this method, total RNA purification, reverse transcription, and PCR amplification are not required, and target mRNA is thus directly detected in cytolysis solutions. Data were corrected by *Pp1a* levels.

#### Cosine curve fitting of experiment data

Acrophases were calculated using a software called Acro, which is provided by Dr. Roberto Refinetti.

## Results and discussion

We compared circadian clock gene expression during a period of habitual nighttime exercise with that during a no-exercise period (Table 1). The physical exercise performed by the subject involved a type of combat sport, and is quite intense. In this experiment, hair follicle cells were collected from the chin every four hours around the clock. The advantages of using hair follicle cells are that they can be obtained relatively non-invasively and fresh naked cells could be obtained simply by plucking hairs without additional cell separation. Importantly, these cells are suitable for the isolation of high-quality total RNA [13]. In the present experiments, gene expression was detected using a branched DNA (bDNA)-based assay. In our previous examination of the circadian expression of 7 clock genes, we found that while the expression of *Per3*, *Nr1d1/Rev-erbα*, and *Nr1d2/Rev-erbβ* genes exhibited clear and well-reproducible circadian fluctuations, the *Per2* and *Dbp* genes oscillated with lower amplitudes, and were therefore less useful for the detection of circadian properties. For *Bmal1* and *Npas2,* only slight oscillations were detected. We therefore decided to use the *Per3*, *Nr1d1/Rev-erbα*, and *Nr1d2/Rev-erbβ* genes for circadian evaluation in this study.

**Table 1:** Subject information

	**Exercise -**		**Exercise +**
Period	Jul 16 – Aug 31		- Jul 15
Intense exercise	None		20:00 to 22:00
(Total exercise duration)	(0 hrs / day)		(2 hrs / day)
Wakeup time		7.5 ± 0.5	
Meal time		8 ± 0.5	
		12.5 ± 0.5	
		19 ± 0.5	
Bed time		1.5 ± 0.5 (Lights-out time)	
Other exercise		None	

**Figure 1:**
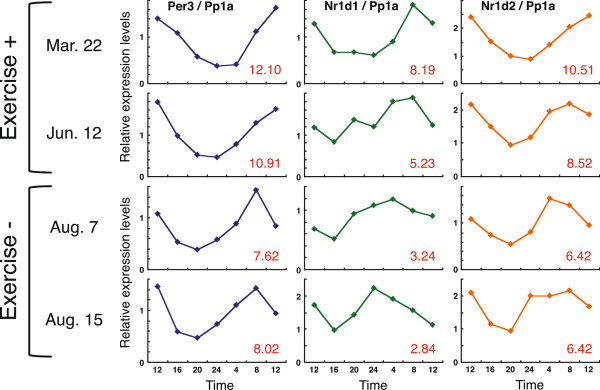
**Effects of excessive nighttime exercise on the phase of circadian gene expression.** In an athlete who regularly performed vigorous exercise between 20:00 and 22:00, the clock gene expression rhythm of facial-hair follicle cells was measured with the branched DNA method; Mar. 22 and Jun. 12 were during the period when the athlete was exercising regularly (Exercise “+”), while Aug. 7 and Aug. 15 were during the period when the athlete was not training (Exercise “-“). Time-course sampling of facial hairs was carried out, and the rhythms of clock gene expression were measured. Clock gene levels were normalized to *Protein Phosphatase 1 A* (*Pp1a*). The lifestyle habits (sleeping and eating) of the subject are shown in Table 1. Acrophases were calculated using the software program Acro, which is provided by Dr. Roberto Refinetti. Calculated circadian peak times are then shown in the right lower corner of each figure (red).

**Figure 2:**
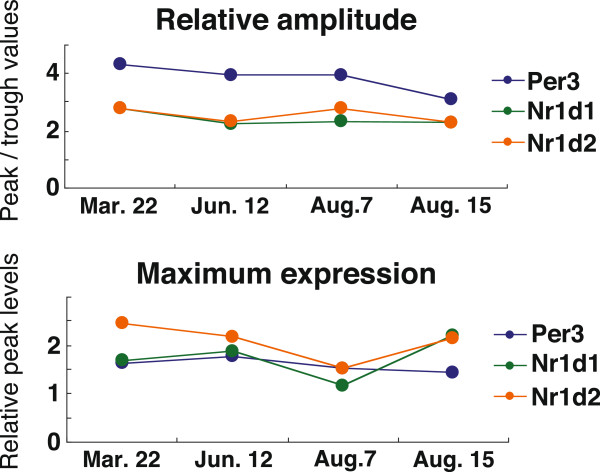
**Effects of excessive nighttime exercise on the amplitude and maximum levels of circadian gene expression.** The relative amplitudes (Top) and maximum levels (Bottom) of clock gene expression rhythms were calculated, and changes over time are shown. After a cosine-curve fitting was performed, relative amplitudes were calculated by dividing peak values by trough values. Maximum levels (Right) are equal to the peak values deduced by the cosine-curve fitting.

We investigated circadian clock gene expression in the subject during a period of hard training at night. 24-h time-course sampling was performed twice during both the exercise and no-exercise periods (“Exercise +” and “Exercise -”), and reproducibility was confirmed (Figure 1). Wake-up and meal times were kept almost constant over the experimental period to reduce the possibility that the data obtained resulted from external factors other than exercise. In the off period (Exercise -), *Per3*, *Nr1d1/Rev-erbα* and *Nr1d2/Rev-erbβ* were expressed in circadian fashion with a phase similar to those in subjects who have a regular lifestyle [13]; generally, the expression peak of these three clock genes is observed just before or a few hours before wake-up time. The time-series data fit a cosine curve well, and the phase relationship between *Per3*, *Nr1d1/Rev-erbα* and *Nr1d2/Rev-erbβ* was well reproducible. Acrophases were calculated using a software called Acro, which is provided by Dr. Roberto Refinetti. During the period in which nighttime training (from 20:00 to 22:00) was habitually performed (shown as “Exercise +”), circadian clock gene expression was clearly phase-delayed by 2 to 4 h compared with that in “Exercise –”. This delayed phase was observed in the circadian expression of all clock genes examined; the expression peak of these genes was detected just after or a few hours after wake-up time. Although PER3, NR1D1, and NR1D2 are not components of the classical core negative feedback loop, the phase relationship between these three genes and *Per2* expression rhythms has been well reproducible among almost all subjects we have examined to date. Even under phase advance lifestyle conditions, the phase relationship was basically maintained before and after the phase shift, suggesting that these three genes are also reliable markers of the core clock machinery. The maximum level and circadian amplitude of clock gene expression were not clearly affected by nighttime training (Figure 2). We thus confirmed that the intense exercise affected phase only among the waveform parameters of circadian gene expression. Although preliminary, these observations illustrate the possibility that heavy nighttime physical exercise might affect circadian phases of clock gene expression. Further, they suggest that athletes might need to schedule heavy physical exercise carefully in order to maintain normal circadian clock function and ensure optimal athletic performance.

The present data provide preliminary but promising evidence indicating that habitual intense exercise affects circadian phase. We are presently unable to explain the mechanism of exercise-induced phase shift seen here. However, based on previous reports, we speculate two possible explanations. First, exercise increases body temperature, which could in turn phase-shift circadian gene expression. Indeed, it was reported that environmental temperature could change circadian phase of core body temperature and phase-shift circadian gene expression in the livers of mice [14]. Second, intense exercise stimulates cortisol secretion, which would directly or indirectly induces circadian phase shift. Glucocorticoid strongly acts on peripheral clocks in mice [15]. In addition to these explanations, exercise might also affect concentrations of other blood-borne factors, which would then trigger circadian entrainment.

The present study might indicate that, since wrong circadian timing of intense exercise might be a disturbing factor against the circadian clockwork, introduction of the concept of circadian timing into training and exercise regimens might of significance for athletes. Furthermore, intense exercise might be a promising factor in the clinical care of circadian disorders and jet lag.

## Conclusion

Although still preliminary, the present trial suggests the possibility that heavy physical exercise might strongly affect the circadian phase of clock gene expression. Exercise regimens might therefore be useful for the clinical care of circadian disorders. On the other hand, the data raise concerns that athletes may require careful scheduling of heavy physical exercise to ensure normal circadian phase and optimal athletic performance.

## Competing interests

The authors declare that they have no competing interests.

## Authors’ contributions

AO performed gene expression assays. TY collected and analyzed data. RM assisted with data collection and analysis. KN assisted with drafting manuscript. MA conceived of the project, supervised gene expression assays and drafted the manuscript. All authors approved the final manuscript.
